# Prevalence of Congenital Anomalies: A Community-Based Study in the Northwest of Iran

**DOI:** 10.1155/2014/920940

**Published:** 2014-03-26

**Authors:** Hossein Mashhadi Abdolahi, Mohammad Hassan Kargar Maher, Farzaneh Afsharnia, Saeed Dastgiri

**Affiliations:** ^1^Tabriz Health Services Management Research Centre, Tabriz University of Medical Sciences, Tabriz 5155668474, Iran; ^2^Pediatric Health Research Centre, Tabriz University of Medical Sciences, Tabriz 5156734511, Iran; ^3^Tabriz District Health Centre, Tabriz University of Medical Sciences, Tabriz 5143814998, Iran

## Abstract

*Background*. Congenital anomalies are responsible for a remarkable proportion of mortality and morbidity in newborns. The aim of this study was to document the epidemiological features of congenital anomalies in rural areas, northwest of Iran. *Method*. The study population included live births born between 2004 and 2012 in rural areas of Tabriz district. All health records of the children under 8 years were assessed retrospectively. *Results*. Of 22500 live births, 254 cases were identified with a primary diagnosis of congenital anomalies giving a prevalence rate of 112.89 per 10 000 births (95% CI: 99.08 to 126.69). Anomalies of the nervous system were the most common defects, accounting for 24% of birth defects followed by the heart diseases anomalies. The highest prevalence rate for birth defects was observed in the south-western region with 386 per 10 000 births (95% CI: 215 to 556) compared to the similar rate in the north-western region with 15 per 10 000 births (95% CI: −14 to 45). *Conclusion*. The considerable geographic disparities in the prevalence of congenital anomalies in the region might be attributed to the highly polluted industrial zone in the area (including air and water pollution, etc.). This needs further etiological investigations in the region.

## 1. Introduction

Congenital anomalies affect a remarkable proportion of newborn population and contribute significantly to the childhood mortality and hospital admissions [1]. Congenital anomalies are a global health problem. Every year an estimated 7.9 million children are born with a serious birth defect, 3.3 million children (under five years) die from birth defects, and 3.2 million who survive may develop a disability later in the life [2]. They are the leading cause of prenatal mortality and childhood morbidity and disability in many countries [1]. The wide range of causes of birth defects means that a portfolio of prevention approaches is needed. The prevention of these disorders is available in 60% of cases [3, 4]. This needs however epidemiological information.

Prevalence studies of congenital anomalies are useful to establish baseline rates, to document changes over time, and to identify clues to the etiology. Many of developed countries monitor the prevalence of birth defects through registration or surveillance system of fetuses and infants. In addition, international organizations have been established to conduct worldwide surveillance and research into the occurrence and possible causes of congenital anomalies and to establish prevention strategies [5].

Congenital anomalies are the most common causes of death in children (1–59 months) in Iran [6]. Studies reporting birth defects prevalence in Iran, however, are mostly limited to particular type of defects [7–11]. Without comprehensive data on congenital anomalies, it is difficult to evaluate possible teratogens and to implement effective prevention and care services. This information is also important for planning and performing antenatal screening for congenital anomalies, particularly in high risk populations. Nevertheless, published comprehensive data about the prevalence of birth defects are scarce in developing countries including Iran. The aim of this study was to determine the epidemiological features of congenital anomalies in rural areas of Tabriz, a major city in the northwest of Iran.

## 2. Methods

Rural Iran benefits from a well-established primary health care (PHC) network. The network is well organized and is credited with the improvements in health outcomes that have been observed since the 1980s in rural areas [12]. The health houses are the first level of contact between the rural community and the health network in the country covering one or several villages in a region. Each health house is staffed by one or more Behvarzes who recruited permanently by the Ministry of Health and usually come from the health houses catchment area. Before Behvarzes start working in health houses, they attend a special two-year training program. Some of the important tasks of Behvarzes are annual census of the population covered, prenatal, natal and postnatal care, care of children under 5 years of age, care of school age children, family planning services, immunization, case finding and referral, and home visits for followup of drop-out cases. They also serve as an important component of the health information system of the PHC network. The system gathers all health related data and provides health information in health houses catchment area. Since each Behvarz covers about 1500 people and s/he is well anchored in her/his community, the risk for missing data is very low. All health cares provided by health houses are free of charge. Rural health centers are headed by physicians and staffed by paramedical and administrative employees. They support and supervise the activities of the rural health houses in the catchment area.

This study was carried out in the district of Tabriz, a major city in the northwest of Iran. The study population comprised live births born between 2004 and 2012 in rural areas of Tabriz district. The district of Tabriz has the largest population in the East Azerbaijan province. Rural population in the region is about 158731 people receiving their primary health care from 47 health houses and 17 health centers.

Congenital anomalies were defined as structural defects, chromosomal abnormalities, inborn errors of metabolism, and hereditary disease diagnosed before, at, or after birth [13].

All 22500 health records of the children (live births) under 8 years of age in the health houses were assessed by 10 expert health workers, and the children with confirmed congenital anomalies were identified. Nearly all births regardless of the birth place are routinely recorded in rural areas. Each child has a health file including her/his health related life events. The coverage for recorded information is nearly complete for those under 8 years of age. The definition of the congenital anomalies was based on the standard coding system of the International Classification of Diseases (10th edition) according to the primary diagnosis of anomaly. For each case, basic demographic information and a detailed clinical description of the birth defect(s) were collected.

For inclusion in the study, a live birth (being alive or deceased) must have been born between 2004 and 2012 and have had health records in the health houses of the rural areas of Tabriz at the time of the study. Total prevalence was calculated by dividing the numerator (registered cases of congenital anomalies) by the relevant denominator (total live births) for the same period of time. A child with more than one anomaly was counted once only based on the primary diagnosis. We calculated 95% confidence interval (CI) for each prevalence rate. All tables and figures were generated using Microsoft Excel 2010.

The study obtained ethics approval from the committee of ethics in Tabriz University of Medical Sciences.

## 3. Results

Out of 22500 live births in rural Tabriz, 254 were diagnosed as having congenital anomalies giving a total prevalence rate of 112.89 per 10 000 live births (95% CI: 99.08 to 126.69).

Baseline demographic characteristics of cases including gender, life status, mother age at pregnancy, and birth order are presented in Table 1. Gender distribution of the birth defects was 59% (150 cases) in male, 41% in female (104 cases) representing a sex ratio (M/F) of 1.44. Of 254 children diagnosed with birth defects, 82 had died due to different reasons and 172 survived.

**Figure 1:**
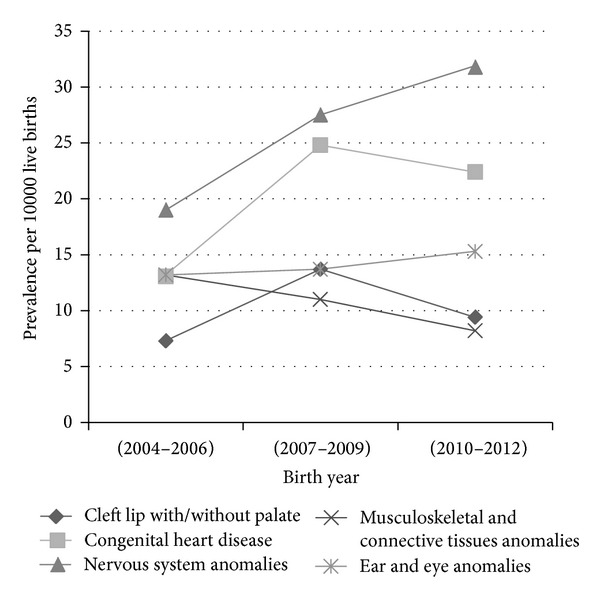
Total prevalence for selected groups of congenital anomalies by year in Tabriz, Iran.

Table 2 shows the prevalence of the main categories of congenital anomalies in the area. Anomalies of nervous system, congenital heart diseases, and ear/eye defects accounted proportionally for more than 55% of anomalies in the region. By contrast, categories of digestive system anomalies, genitourinary tract, and defects of respiratory system accounted all for less than 10% of anomalies.  Table 3 and Figure 1 show the time trends for selected groups of anomalies. The prevalence of anomalies of nervous system increased from 19 per 10 000 births (95% CI: 8.7–29.5) in 2004–2006 to 31.9 (95% CI: 19.9–43.9) in 2010–2012. Ear and eye defects also showed an upward trend from 9 per 10 000 births (95% CI: 8.7–29.5) in 2004–2006 to 15.3 (95% CI: 7–23.7) in 2010–2012.

Of 22 children with defects of the neural system, 12 had microcephaly, eight had hydrocephaly, and two had macrocephaly. Nine subjects of birth defects with cleft lip were accompanied with cleft palate. Only 17 cases of congenital anomalies were associated with a chromosomal anomaly.

The highest prevalence rate for birth defects was observed in the south-western region with 386 per 10 000 births (95% CI: 215 to 556), and the lowest rate was observed in the north-western region with 15 per 10 000 births (95% CI: −14 to 45).

## 4. Discussion

This study was an epidemiological investigation to estimate the prevalence of congenital anomalies in the northwest of Iran.

We found that the total prevalence of birth defects was 113 per 10 000 births while some other studies reported a prevalence rate ranging from 49 to 283 inside the country [14–18], and from 213 to 389 was reported by European Registry of Congenital Anomalies and Twins (EUROCAT) and International Clearinghouse for Birth Defects Surveillance and Research (ICBDSR) for other countries [19, 20].

In this study the most common anomaly was nervous system which is consistent with the similar report from Urmia, a city in the northwest country [15], while congenital heart diseases were reported as the most commonly system affected in EUROCAT region. A study in Yasuj, southwest of Iran, reported a high prevalence of neural tube defects (48 per 10 000 births) in the region [17]. Although musculoskeletal system was the first leading affected system according to a research in Gorgan, northern of Iran [16], it was the fourth one in our study.

The low prevalence of congenital anomalies in this study may be a result of the problems in recording and documentation of the country referral system for primary health care in the rural areas [21].

While in the current study the prevalence of anomalies of nervous system increased from 19 per 10 000 births in 2004–2007 to 32 in 2010–2012, there was a decline in the prevalence of this category in some registries of EUROCAT countries for the years 2000–2009. Unlike ours, EUROCAT statistics reported a downwarding pattern for ear and eye defects in 2000–2009 [22].

The differences between studies might be the effect of different racial, ethnic, and social factors in various parts of the world. Other explanations for these variations in birth defects prevalence could come from study methods (i.e., sampling, criteria for diagnosis, recording, etc.). Moreover, the accessibility and utilization of advanced techniques in developed countries (i.e., fetal visualization using ultrasound screening and chromosome microarray testing at birth) have improved the early detection of anomalies in those countries. This seems also important in explanation of variations between countries.

We found considerable geographic disparities in the prevalence of birth defects in the area ranging between 15 and 386 (per 10 000 births). This may be due to highly polluted industrial zone in the district with high prevalence rates. The southwest of Tabriz district is the most contaminated area in terms of environmental pollution. Tabriz Petrol Refinery, Tabriz Petrochemical Refinery, and Thermal Power Plant as well as Shahid Rajaei Industrial Town are all located in this zone which could be considered as the main sources of pollution in the region.

We had two limitations in this study. First, data came from live births only while in estimating the prevalence of congenital anomalies in population, stillbirths, and pregnancy terminations should be taken into account as well. Second, we lost the data of immigrant families. It means that we did not have access to the health files of families who used to live in rural Tabriz and moved away from this area.

## 5. Conclusion

We conclude that despite the low prevalence of birth defects in the northwest of Iran, the considerable geographic disparities in the prevalence of birth defects in the region might be attributed to highly polluted industrial zone (including air, water pollution, etc.). This needs further etiological investigations in the region.

## Figures and Tables

**Table 1:** Characteristics of the study subjects (Tabriz-Iran, 2004–2012).

	*n*	%
Gender		
Female	104	41
Male	150	59
Life status		
Alive	172	68
Dead	82	32
Birth order		
1	128	50
2	83	33
≥3	43	17
Mother age at pregnancy		
<18	2	0.79
18–35	181	71
>35	71	28.21

**Table 2:** Prevalence rates (per 10 000 live births) of congenital anomalies by type (Tabriz-Iran, 2004–2012).

Congenital anomaly category	Number of anomalies	Rate (per 10 000)	95% confidence intervals
Nervous system anomalies (including NTDs)	60	26.67	(19.92, 33.40)
Neural tube defects (NTDs)	22	9.78	(5.69, 13.86)
Congenital heart diseases	46	20.44	(14.54, 26.34)
Ear and eye anomalies	32	14.22	(9.29, 19.14)
Musculoskeletal and connective tissue anomalies	24	10.67	(6.40, 14.93)
Cleft lip with/without palate	23	10.22	(6.04, 14.39)
Down's syndrome	17	7.6	(3.96, 11.14)
Anomalies of limb	16	7.11	(3.62, 10.59)
Digestive system anomalies	12	5.33	(2.31, 8.35)
Genitourinary tract and kidney	6	2.67	(0.53, 4.82)
Respiratory system anomalies	5	2.22	(0.27, 4.17)
Others	13	5.78	(2.63, 8.91)

Total	254	112.89	(99.08, 126.69)

**Table 3:** Time trends for prevalence rates (per 10 000 live births) of selected groups of congenital anomalies, Tabriz, Iran.

Time trend	Cleft lip with/without palate		Congenital heart diseases		Nervous system anomalies		Musculoskeletal and connective tissue anomalies		Ear and eye anomalies	
	Prevalence	95% CI	Prevalence	95% CI	Prevalence	95% CI	Prevalence	95% CI	Prevalence	95% CI
2004–2006	7.3	0.91–13.8	13.2	4.5–21.9	19	8.7–29.5	13.2	8.7–29.5	13.2	8.7–29.5
2007–2009	13.7	5.2–22.3	24.8	13.3–36.2	27.5	15.4–39.6	11	3.3–18.6	13.7	5.2–22.3
2010–2012	9.4	2.9–16	22.4	12.3–32.5	31.9	19.9–43.9	8.2	2.1–14.4	15.3	7–23.7

## References

[1] Dastgiri S, Sheikhzadeh Y, Dastgiri A (2011). Monitoring of congenital anomalies in developing countries: a pilot model in Iran. *Studies in Health Technology and Informatics*.

[2] Carmona RH (2005). The global challenges of birth defects and disabilities. *The Lancet*.

[3] Czeizel AE (1993). Prevention of congenital abnormalities by periconceptional multivitamin supplementation. *British Medical Journal*.

[4] Czeizel AE, Intody Z, Modell B (1993). What proportion of congenital abnormalities can be prevented?. *British Medical Journal*.

[5] International Clearinghouse for Birth Defects Monitoring Systems (ICBDSR) Annual Report 2005: with data for 2003.

[6] Rahbar M, Ahmadi M, Lornejad HR, Habibelahi A, Sanaei-Shoar T, Mesdeaghinia AR (2013). Mortality causes in children 1–59 Months in Iran. *Iranian Journal of Public Health*.

[7] Rahim F, Ebadi A, Saki G, Remazani A (2008). Prevalence of congenital heart disease in Iran: a clinical study. *Journal of Medical Sciences*.

[8] Nikyar B, Sedehi M, Mirfazeli A, Qourbani M, Golalipour M-J (2011). Prevalence and pattern of congenital heart disease among neonates in Gorgan, Northern Iran (2007-2008). *Iranian Journal of Pediatrics*.

[9] Golalipour MJ, Najafi L, Keshtkar AA (2010). Neural tube defects in native Fars ethnicity in northern Iran. *Iranian Journal of Public Health*.

[10] Farhud D, Hadavi V, Sadeghi H (2000). Epidemiology of neural tube defects in the world and Iran. *Iranian Journal of Public Health*.

[11] Mohammadbegi R, Rahimi E (2002). Neural tube defects innewborns in Bacat hospital of Sannadaj in Kordestan province 6 in Iran. *Scientific Journal of Kurdistan University of Medical Sciences*.

[12] The world health report 2008: primary health care—now more than ever. http://www.who.int/whr/2008/en/.

[13] EUROCAT Working Group (1997). *Eurocat Report*.

[14] Ghahramani M, Moshki M, Ebadi A (2002). A survey of causes and prevalence of congenital anomalies in live born neonates in Gonabad 22 Bahman Hospital. *Ofogh-E-Danesh*.

[15] Abdi-Rad I, Khoshkalam M, Farrokh-Islamlou H (2008). The prevalence at birth of overt congenital anomalies in Urmia, Northwestern Iran. *Archives of Iranian Medicine*.

[16] Golalipour MJ, Ahmadpour-Kacho M, Vakili MA (2005). Congenital malformations at a referral hospital in Gorgan, Islamic Republic of Iran. *Eastern Mediterranean Health Journal*.

[17] Ebrahimi S, Ashkani S, Bagheri F (2013). Prevalence of neural tube defects in Yasuj, Southwest Iran. *Shiraz E-Medical Journal*.

[18] Karbasi SA, Golestan M, Fallah R, Mirnaseri F, Barkhordari K, Bafghee MS (2009). Prevalence of congenital malformations in Yazd (Iran). *Acta Medica Iranica*.

[19] International Clearinghouse for Birth Defects Monitoring Systems (ICBDSR)

[20] EUROCAT Working Group http://www.eurocat-network.eu/prevalencetable.

[21] Eskandari M, Abbaszadeh A, Borhani F (2013). Barriers of referral system to health care provision in rural societies in Iran. *Journal of Caring Sciences*.

[22] http://www.eurocat-network.eu/statisticalmonitoring-2009.

